# Dolutegravir rollout for treatment of HIV with a focus on advanced disease and tuberculosis coinfection: findings from rural KwaZulu-Natal, South Africa (2019–2023)

**DOI:** 10.1186/s12981-025-00810-z

**Published:** 2025-10-24

**Authors:** Reuben Christopher Moyo, Larisse Bolton, Elphas Luchemo Okango, Margot Otto, Nthoesele Letoao, Peter Suwirakwenda Nyasulu, Frank Tanser

**Affiliations:** 1 South African Centre for Epidemiological Modelling and Analysis (SACEMA), School for Data Science and Computational Thinking, Stellenbosch University, Stellenbosch, South Africa; 2 Centre for Epidemic Response and Innovation (CERI), School for Data Science and Computational Thinking, Stellenbosch University, Stellenbosch, South Africa; 3 Department of Pediatrics and Child Health, Faculty of Medicine and Health Sciences, Stellenbosch University, Stellenbosch, South Africa; 4 Africa Health Research Institute (AHRI), Durban, South Africa; 5 Division of Epidemiology and Biostatistics, Department of Global Health, Faculty of Medicine and Health Sciences, Stellenbosch University, Stellenbosch, South Africa

**Keywords:** Tuberculosis, Advanced HIV disease, Dolutegravir, South Africa

## Abstract

**Background:**

While Dolutegravir (DTG) containing antiretroviral therapy (ART) has become the preferred regimen for people living with HIV (PLHIV), the pace and equity of its adoption, especially among subgroups with tuberculosis (TB) symptoms and advanced HIV disease (AHD), remain understudied in high-burden settings like rural KwaZulu-Natal (KZN), South Africa. This study describes the transition to DTG and examines the effect of TB and AHD (CD4 count < 200 cells/mm^3^) on the likelihood of transitioning to DTG in rural KZN, South Africa.

**Methods:**

We conducted a longitudinal cohort analysis using routine HIV program data from 69,916 PLHIV aged ≥ 15 years attending 19 HIV clinics in rural KZN, between 1st October 2019, and December 31st 2023. Kaplan-Meier analysis estimated time to DTG transition, while a multivariate mixed-effect Cox proportional hazards model evaluated factors associated with transitioning to DTG.

**Results:**

Of the 69,916 PLHIV included in the cohort, 49,365 (70.6%) were female, and the median age of the PLHIV was 40 years (IQR: 32–49). By the end of the follow-up period, 70.9% (*n* = 49,598) of the PLHIV transitioned to DTG in 165,880 person-years. The median time to DTG transition was 14 months among PLHIV without TB symptoms, compared to 22 months among those with TB symptoms. Similarly, PLHIV with CD4 counts ≥ 350 cells/mm³ transitioned at a median of 14 months, while those with CD4 < 200 cells/mm³ transitioned 14 months later. The likelihood of transitioning to DTG was 22% lower among PLHIV with TB symptoms (aHR = 0.78, 95% CI: 0.76, 0.82) compared to their counterparts without TB symptoms, and 43% lower among PLHIV with AHD (aHR = 0.57, 95% CI: 0.54,1.59) compared to their counterparts without AHD.

**Conclusion:**

Our analysis showed that over a quarter of the PLHIV in rural KZN remained on non-DTG-containing regimens by 31st December 2023. PLHIV coinfected with TB and having AHD transitioned at a slower pace than their counterparts.

## Background

In South Africa, the ART program has primarily relied on Efavirenz (EFV), a class of non-nucleoside reverse transcriptase inhibitor (NNRTI), as its main component since 2004 [1]. However, the management of PLHIV using EFV-based regimens as the main component of ART was increasingly undermined by the NNRTI drug-resistant HIV strains [2, 3]. In 2018, the World Health Organisation (WHO) recommended DTG, an integrase strand transfer inhibitor (INSTI) class of ART [4], as an alternative to EFV. DTG was preferred because of its high genetic barrier to resistance [2, 3], improved tolerability [5], superior efficacy [6], and greater cost-effectiveness [7] compared to EFV-based regimens. South Africa started transitioning to DTG in September 2019 [2].

TB, a highly infectious disease caused by Mycobacterium Tuberculosis that primarily affects the lungs, is one of the leading causes of death among PLHIV [8]. In 2023, approximately 10.8 million people were diagnosed with TB globally, resulting in 1.25 million deaths, including 161,000 in PLHIV [8]. South Africa has one of the world’s highest TB burdens, with an estimated incidence rate of 468 per 100,000 person-years, of which a substantial proportion occurs in PLHIV [9]. The first South African national TB survey conducted in 2018 reported a TB prevalence of 852 (95% CI: 679,1026) cases per 100,000 population [10]. The survey further reported a prevalence of 1,734 (95% CI: 1219,2234) cases per 100,000 population among PLHIV. TB is common among PLHIV with AHD [11], which is considered a late stage of the HIV infection, characterised by severe immune system damage [12].

The WHO defines AHD as a CD4 count < 200 cells/mm^3^ in PLHIV or the presence of WHO clinical stage 3 or 4 conditions, such as pulmonary TB, oral Candidiasis, unexplained weight loss of >10% of body weight, and chronic diarrhoea lasting more than a month in adults and adolescents [13]. In South Africa, AHD continues to represent a major public health concern, despite the country’s large and widely accessible ART program [14]. A significant proportion (20%) of PLHIV newly initiated on ART and those re-entering care after treatment interruption present with AHD [15]. PLHIV with AHD face elevated risks of opportunistic infections, including TB, cryptococcal meningitis, and pneumocystis pneumonia, all of which contribute to high morbidity and mortality rates [16–18]. Between 2008 and 2021, the proportion of patients presenting with AHD dropped from approximately 62% to 20%, reflecting expanded access to testing and early ART initiation [19]. Early initiation of ART and prompt TB treatment in PLHIV has been shown to offer improved HIV-TB clinical outcomes [20].

Evidence on the overall transition to DTG, and how this transition differs by TB disease status, which is a leading cause of death among PLHIV and AHD, is sparse. Knowing which categories of PLHIV have not transitioned to DTG, including those coinfected with TB, would be useful in addressing patient or clinical-related reasons for not transitioning to DTG. Gaining insight into these barriers can inform targeted interventions to promote a more widespread adoption of DTG-based regimens as well as future ART regimens. This study described the transition to DTG and examined the effect of TB and AHD on the likelihood of transitioning to DTG using HIV program data from October 1st, 2019, to December 31st, 2023, in rural KwaZulu-Natal, South Africa.

## Methods

### Study design and setting

This was a prospective cohort study design, utilising routinely collected deidentified HIV program data from 19 HIV clinics under the Hlabisa HIV treatment and care programme in Umkhanyakude, rural KZN, South Africa [21]. The HIV incidence in the study setting is high, estimated at 2.5 per 100 person-years for females and 1.5 per 100 person-years for males [22]. The Hlabisa HIV treatment and care programme, established in 2004, enrolls and tracks PLHIV, enabling detailed monitoring of ART uptake, retention, and programmatic shifts such as the rollout of Dolutegravir [23].

### Data sources

The study used clinical program data from the three interlinked electronic registers (TIER.NET), an HIV data capturing and management system introduced in South African clinics in 2010 [24]. PLHIV who started ART before the introduction of TIER.NET had their data transferred into TIER.NET from previous ART databases and patient master cards.

### Measures

The following variables were considered in our analyses: (1) Regimen type: This variable described whether an individual was on a DTG-containing regimen or a non-DTG-containing regimen. This variable was created by grouping all regimens containing DTG into the DTG-containing regimen group and all other regimens without DTG into the non-DTG-containing regimen group (Those on EFV-based regimens or Protease inhibitors (PIs)). (2) Viral load suppression (VLS): This time-varying variable showed whether an individual was virally suppressed or not during the follow-up period. This variable was created by using a cut-off point of ≤400 copies per millilitre of blood, while those with >400 copies per millilitre of blood were considered not suppressed [25, 26]. (3) TB disease status: This variable describes whether an individual developed active TB during or not. Information on TB status was found in TB registers of the PLHIV who were found with clinical symptoms consistent with TB and were put on TB treatment. (4) CD4 count: CD4 count, measured in cells/mm³, serves as an indicator of immune function and therefore disease progression in PLHIV. We categorised CD4 count into four groups: 0–199, 200–349, 350–499, and ≥ 500 cells/mm³. CD4 was observed at different observation dates, making it time-varying because its values changed during follow-up. PLHIV with a CD4 count < 200 cells/mm^3^ were classified as having AHD. (5) Sex of participants: This variable described the biological differentiation of sex into males and females, as documented in TIER.NET. (6) Participant age groups: Age groups were generated from the participants’ age variable, generated by subtracting the observation date from the participant’s date of birth, to create age at different observation dates, which were then categorised. Age of the PLHIV was categorised into the following categories: 15 to 24, 25 to 34, 35 to 44, 45 to 54, and above 55 years.

### Inclusion and exclusion criteria

**Fig. 1:**
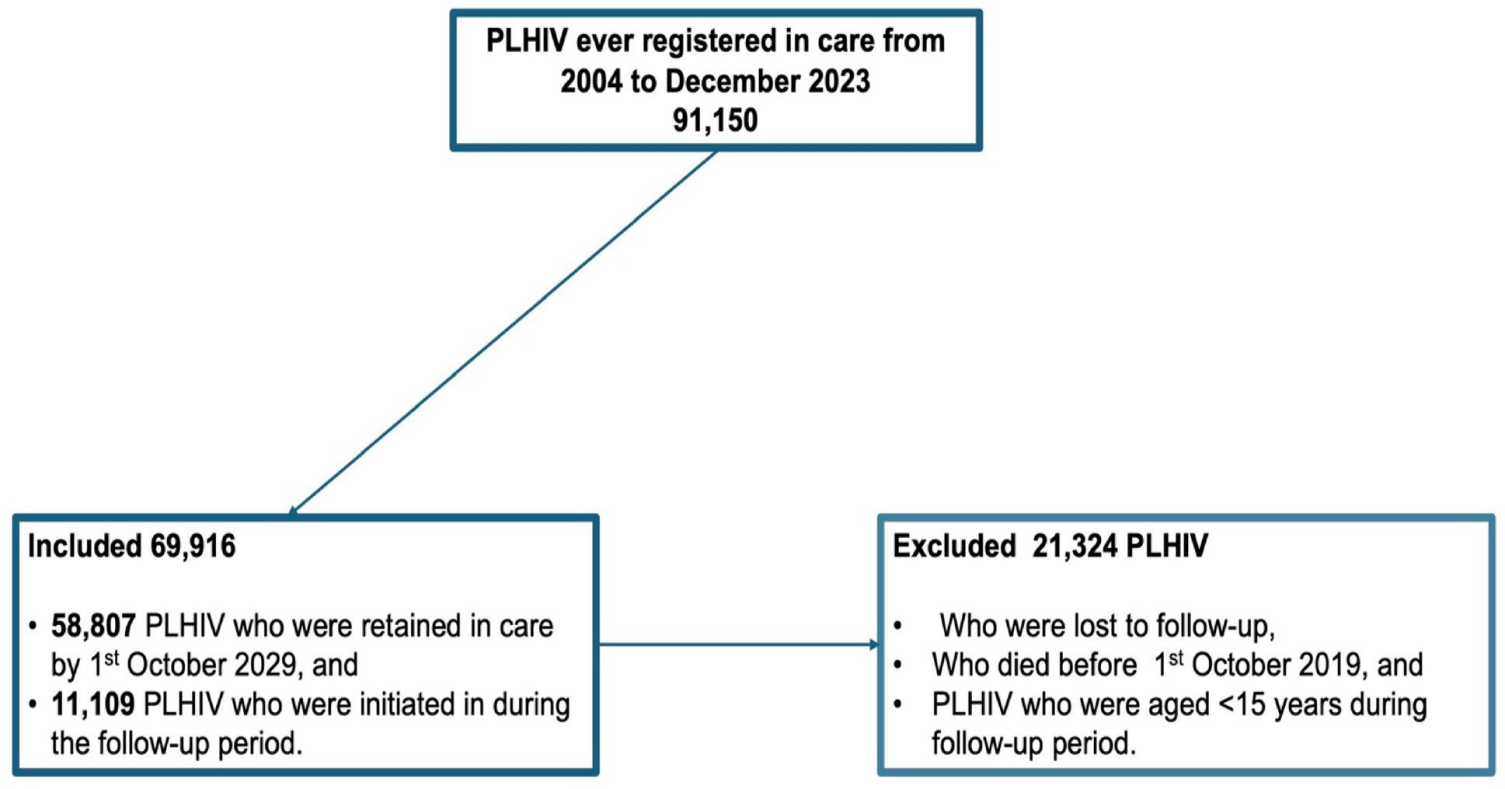
Sample size flow diagram

Figure 1 is a flow diagram of the sample size of the cohort used in our analysis. We included PLHIV aged 15 and above who were still in care by 1st October 2019 and who were registered in care between 1st October 2019 and 31st December 2023. We excluded PLHIV aged below 15 because of the limited paediatric DTG formulation at the beginning of the transition. We also excluded PLHIV who died or were lost to follow-up before 1st October 2019. PLHIV who were still in care at the beginning of the follow-up were 58,807, of whom 70.2% were female. PLHIV who were registered in care during the follow-up period were 11,109, of whom 66.0% were female. By the end of follow-up, a total of 69,916 were included in the study.

### Statistical analysis

Descriptive statistics were used to characterise baseline participants’ characteristics, such as TB status, age groups, regimen type, CD4 count, and sex, using frequencies and percentages. Because PLHIV transitioned to DTG at varying time points during follow-up, we applied Kaplan-Meier methods to describe transition patterns by TB status, age groups, sex, and CD4 count [27]. We evaluated factors associated with the likelihood of transitioning to DTG using a multivariate mixed-effects Cox proportional hazards model, accounting for clustering by clinic. The Cox proportional hazard models are distribution-free survival models that assume that the survival/failure curves for two or more strata of the predictor variables are proportional over time [28], and have been widely used in public health research to fit models for time-to-event data. Since the CD4 count and viral load data had missing values, we applied regression-based multiple imputation to impute the missing observations for these covariates. Multiple imputation is the recommended approach for missing information in longitudinal survival data [29, 30]. Covariates evaluated in the final model were: sex, age groups, VLS, TB status, and CD4 count.

## Results

Of the 69,916 PLHIV included in the cohort from 1st October 2019 to 31st December 2023, 49,365 (70.6%) were female, and the median age of the PLHIV was 40 years (IQR 32–49 years). Table 1 shows the distribution of baseline characteristics of the PLHIV. Clinical symptoms consistent with TB at baseline were present in 1.6% of the PLHIV, while AHD was present in 14.5% of the PLHIV. In terms of virologic outcomes at baseline, 64.1% of the PLHIV were virally suppressed. At the beginning of follow-up, only 1.5% of the PLHIV were on DTG-containing regimens.

**Table 1:** Description of baseline characteristics of the PLHIV (*N* = 58,807)

Characteristic	Frequency	Column %
*Sex*		
Male	17,524	29.8
Female	41,283	70.2
*Age group*		
15–24	3,035	5.2
25–34	12,148	20.7
35–44	20,353	34.6
45–54	13,436	22.9
55 and above	9,835	16.6
*CD4 categories*		
< 200	8,533	14.5
200 to 350	10,932	18.6
351 to 499	11,950	20.2
500 and above	27,392	46.8
*Viral load suppression*		
No	21,112	35.9
Yes	37,695	64.1
*TB disease symptoms*		
Symptomatic	941	1.6
Asymptomatic	57,866	98.4
*Regimen type*		
DTG containing	882	1.5
Non-DTG containing	57,925	98.5

Baseline characteristics were based on the PLHIV who were still in care at the beginning of the follow-up on 1st October 2019

**Table 2:** Cox proportional hazard model of the hazard to transition to DTG among PLHIV in rural KZN, South Africa—1st October 2019 to 31st December 2023

	Adjusted hazard ratio (95% CI)	*p*-value
*On TB treatment*		
No	1	
Yes	0.78 (0.76–0.82)	0.001
*Sex*		
Male	1	
Female	1.05 (0.92–1,19	0.468
*Age group*		
15–24	1	
25–34	1.30 (1,23–1.25)	< 0.001
35–44	1.29 (1.26–1.31)	< 0.001
45–54	1.31 (1.29–1.33)	< 0.001
55 and above	1.38 (1.36–1.41)	< 0.001
*VLS*		
No		
Yes	1.33 (1.31–1.34)	< 0.001
*CD4 count category*		
< 200	0.57 (0.54–1.59)	< 0.001
200 to 350	0.70 (0.68–0.72)	< 0.001
351 to 499	1	–
500 and above	0.94 (0.92–0.96)	< 0.001

**Fig. 2:**
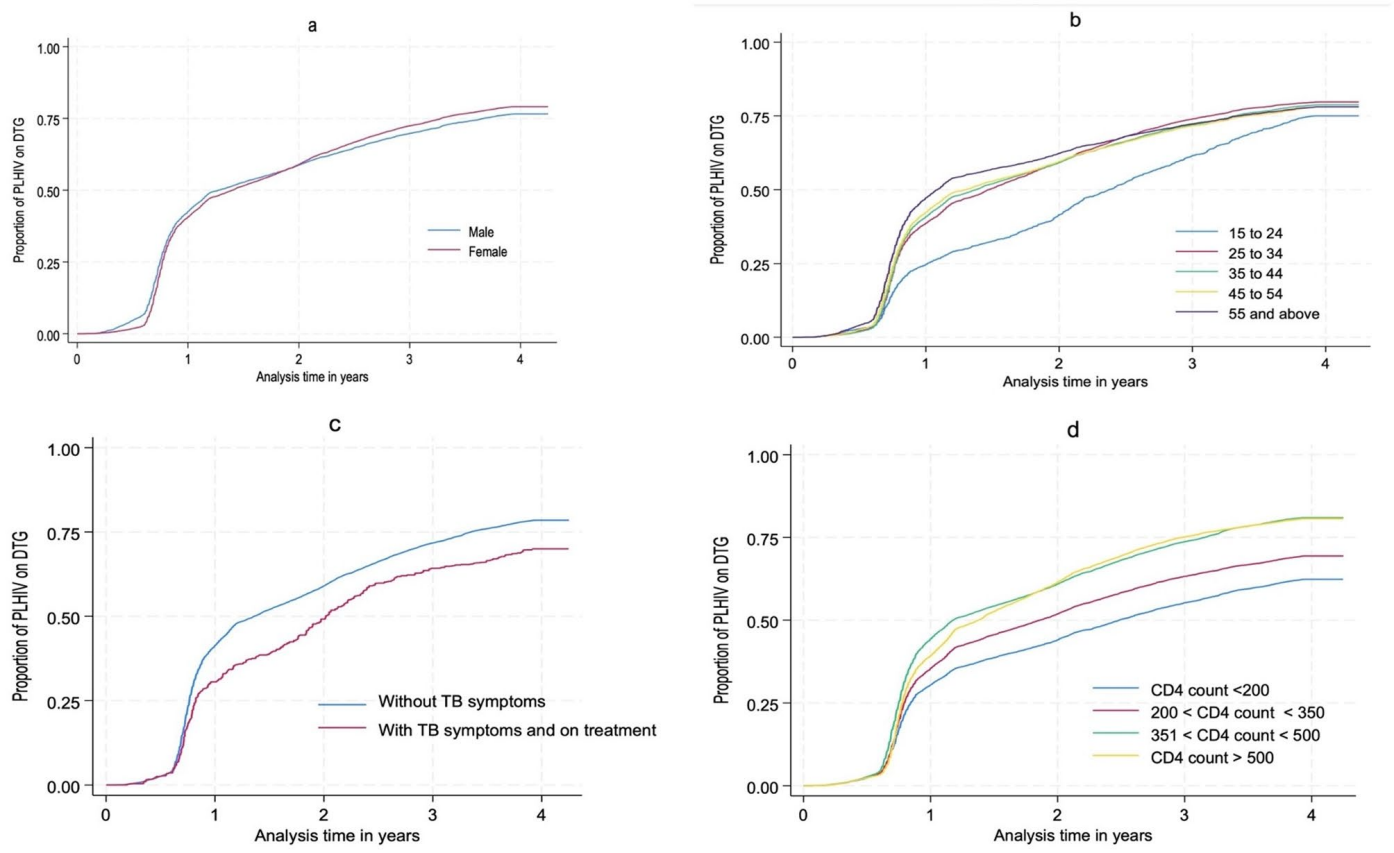
Proportion of PLHIV transitioning to DTG over time. This figure indicate the proportion of PLHIV transition to DTG by Sex (**a**), age groups (**b**), TB status (**c**), and CD4 count (**d**)—1st October 2019 to 31st December 2023

By 31st December 2023, we observed 48,598 (70.9%) PLHIV transitioning to DTG in approximately 165,880 person-years of observation, whereas 21,318 (29.1%) remained on non-DTG containing regimens. While overall DTG transition showed minimal gender disparity, more females transitioned to DTG than males beginning in 2021. Kaplan-Meier curves (Fig. 2a, d) of the transition to DTG indicate that the transition is delayed in younger PLHIV, particularly those aged 15 to 24 years (Fig. 2a), compared to older PLHIV. The median time to DTG transition among PLHIV without symptoms of TB was 14 months, while that of PLHIV with TB symptoms was observed 8 months later. The median time to DTG transition among PLHIV with CD4 count > 350 was 14 months, while those with CD4 < 200 cells/mm^3^ were observed 14 months later. The transition to DTG was persistently lower across analysis time among PLHIV with active TB symptoms (Fig. 2c) and those with AHD (Fig. 2d).

Regarding the likelihood of DTG transitioning (Table 3), the hazard of transitioning to DTG was 22% lower among PLHIV with TB symptoms compared to those without TB symptoms (aHR = 0.78, 95% CI: 0.76,0.82). Age was positively associated with DTG transition. Compared to PLHIV aged 15 to 24 years, the likelihood of transitioning to DTG among PLHIV aged 25 to 34 and ≥ 55 years was 30% higher (aHR = 1.30, 95% CI: 1.23, 1.38) and 38% higher (aHR = 1.38, 95% CI: 1.36, 1.41), respectively. VLS was also a strong predictor of transitioning to DTG; the likelihood of transitioning to DTG was 33% higher among PLHIV who were suppressed (aHR = 1.33, 95% CI: 1.31, 1.36) compared to their counterparts who were not suppressed. In contrast, low CD4 count significantly reduced the likelihood of transition; the likelihood of transitioning to DTG among PLHIV with AHD was 43% lower hazard (aHR = 0.57, 95% CI: 0.54, 0.58) compared to those with CD4 counts between 351 and 499 cells/mm³.

## Discussion

This study described the transition to DTG and evaluated factors associated with the likelihood of transitioning to DTG in rural KZN, South Africa, between 1 st October 2019 and 31 st December 2023. Our findings indicate that transition to DTG was incomplete, with over a quarter of the PLHIV in the study area remaining on EFV-based regimens. Furthermore, our findings indicate the transition to DTG lagged in PLHIV coinfected with TB, those 15 to 24 years, and PLHIV with AHD. By 31 December 2023, the transition to DTG was 19 percentage points below the 90% target set by the South African National Department of Health [31].

Overall, the transition to DTG in South Africa lags behind that of other sub-Saharan African countries. In Malawi, the target for DTG transition was achieved in 2021 [32], while in Zimbabwe, approximately 80% of PLHIV transitioned to DTG by 2021 [33], and 98% of the PLHIV in Zambia transitioned to DTG by 2023 [34]. Despite our study setting bearing/reflecting the high burden of HIV [19], limited training among service providers during the early stages of the transition on DTG interactions with other drugs [35], may have contributed to the slow pace. The lagged transition in PLHIV with TB symptoms could have been attributed to concerns about drug–drug interactions with Rifampicin, an antibiotic used as the main component of TB treatment [36]. Evidence indicates that Rifampicin interacts with DTG, reducing its plasma levels and consequently, its efficacy [37, 38]. Evidence from randomised controlled trials indicates that a double dose of DTG in PLHIV coinfected with TB and receiving Rifampicin results in favourable clinical outcomes due to immune recovery [38]. The current South African National Department of Health HIV - TB treatment guidelines were subsequently amended to guide the management of HIV-TB coinfection [39].

For PLHIV with AHD, the slow transition may reflect concerns about potential adverse effects of DTG, including immune reconstitution inflammatory syndrome (IRIS), neuropsychiatric symptoms, and adherence challenges [40, 41]. At the beginning of the transition, the Southern African HIV Clinicians Society recommended switching patients with viral load < 50 copies/ml from NNRTI-based regimens to DTG while keeping the same Nucleoside Reverse Transcriptase Inhibitors (NRTIs) backbone. For PLHIV with viral load ≥50 copies/ml, they advised delaying the switch, providing adherence support, and repeating viral load testing. If the target viral load was not achieved, patients were transitioned to second-line DTG or protease inhibitors (PIs)-based regimens with adjusted NRTI backbones based on resistance [42]. This guidance may partially explain the delayed transition observed in PLHIV with high viral loads and low CD4 counts.

Accelerating the transition to DTG among PLHIV with AHD could also improve their clinical outcomes, including reducing the risk of TB [11]. This study utilised data from a setting with high HIV burden and high rates of TB infection to assess progress in the transition to DTG [43, 44]. It is also one of the few studies to examine how demographic and clinical factors contribute to disparities in DTG scale-up. These findings highlight the need for targeted interventions to accelerate the delayed transition to DTG among PLHIV coinfected with TB or with AHD to improve clinical outcomes in rural KZN. Strategies to improve HIV/TB outcomes, beyond routine clinical monitoring, include but are not limited to: capacitating community care workers to conduct active TB/HIV screening, diagnosis, and linkage to care [45]; delivering integrated home-based TB and HIV treatment to improve adherence, treatment completion, and viral suppression [46]; implementing clinic-level quality improvement programs to optimise patient flow, data review, and proactive identification of treatment-eligible individuals [47]; and expanding integrated community-based case finding and preventive therapy campaigns to reduce risk of transmission, disease progression, and late presentation [48].

This study has limitations. First, it may be subject to provider bias in decisions regarding which individuals were switched to DTG, particularly when clinical guidelines were inconsistently followed or if DTG was not readily available at certain times. In addition, misclassification within the clinical data may have introduced bias into the estimates and led to incorrect assumptions regarding the effect of HIV–TB coinfection and AHD on the likelihood of transitioning to DTG. For example, PLHIV on EFV could have been misclassified as being on DTG and vice versa, or PLHIV without TB symptoms could have been recorded as having TB symptoms and vice versa. However, such misclassifications were minimised in our study by verifying regimen type at each visit and cross-checking TB status in TB registers. Differential loss to follow-up could be another source of bias, particularly if PLHIV who were lost to follow-up were more likely to transition to DTG than those who remained in care. However, the loss to follow-up observed in this study was minimal and unlikely to substantially bias our estimates [49].

### Conclusion

Our analysis showed that by 31st December 2023, over a quarter of the PLHIV in rural KwaZulu-Natal remained on non-DTG-containing regimens. Those coinfected with TB or having AHD transitioned at a slower pace than their counterparts. The findings highlight the need to prioritise key groups, such as PLHIV who are coinfected with TB or have AHD, to ensure successful transition to DTG-containing regimens.

## Data Availability

Data for this study are freely available and requested from the Africa Health Research Institute (AHRI) by contacting the data management officer.
